# Left atrial myxoma with cardiogenic shock following a myocardial infarction: a case report

**DOI:** 10.1186/s13256-024-04420-7

**Published:** 2024-03-11

**Authors:** Ian Lancaster, Tara Hrobowski-Blackman, Deep Patel, Lubna Chatoor, Joshini Simon, Andrew Willinger

**Affiliations:** HCA Healthcare/USF Morsani College of Medicine GME Programs, Largo Medical Center, 201 14Th St SW, Largo, FL 33770 USA

**Keywords:** Myxoma, Acute mitral dynamic stenosis, Cardiogenic shock, Myocardial infarction, Obstruction, Case report

## Abstract

**Background:**

Myxomas are the most common primary cardiac tumor and typically originate in the left atrium. Atrial myxomas may present following complications of obstruction and emboli. If an atrial myxoma goes untreated, complications such as congestive heart failure, embolic stroke, and sudden death can occur.

**Case presentation:**

A 58-year-old Caucasian male presented following a cardiac arrest. He was taken emergently to the cardiac catheterization lab and received two drug eluting stents. Following the procedure, he was found to have a left atrial mass that was intermittently obstructing the mitral valve on echocardiography. After leaving the cardiac catheterization lab, he was hypotensive and placed on multiple intravenous medications for hemodynamic support as well as an Impella device. Following medical optimization, he underwent one vessel coronary artery bypass graft as well as surgical excision of the left atrial mass, which pathology had shown to be an atrial myxoma.

**Conclusion:**

This patient’s case of cardiogenic shock following revascularization was complicated by the identification of an atrial myxoma, which, when large enough, can obstruct blood flow through the mitral valve leading to acute mitral dynamic stenosis. This condition results in circulatory collapse due to obstruction of the left ventricle in diastole as the myxoma occludes the mitral valve.

## Background

Myxomas are the most common primary cardiac tumor [1]. More than 70% of all cardiac myxomas originate from the left atrium and 18% from the right atrium [2]. Most myxomas present with either manifestations of embolic or obstructive complications [2]. These tumors typically arise from the interatrial septum at the border of the fossa ovalis, but they can also originate from the posterior atrial wall, anterior atrial wall, and the atrial appendage [2]. Myxomas typically appear as a mobile mass attached to the endocardial surface by a stalk, usually arising from the fossa ovalis [2].

Myxomas usually present with one or more of the classic triad of symptoms of (1) intracardiac obstruction (pulmonary edema, dyspnea, orthopnea, malaise, syncope, and palpitations), (2) embolic signs (to the central nervous system, to the peripheral arteries, or to the coronary arteries), and (3) constitutional symptoms (fever, weight loss, and fatigue) [1]. Notably, left atrial myxomas typically present with systemic or peripheral embolization [1]. If an atrial myxoma goes untreated, complications such as congestive heart failure, embolic stroke, and sudden death can occur [3]. Surgical resection is the definitive treatment. In this case report, we discuss the case of a 58-year-old Caucasian male who presented following cardiac arrest and who was ultimately found to have significant coronary artery disease as well as large left atrial myxoma. In this case report, we will focus our discussion on the etiology, presentation, treatment, and management of left atrial myxomas.

## Case presentation

A 58-year-old Caucasian male presented via emergency medical services (EMS) following a cardiac arrest. He was found in ventricular fibrillation by EMS, resuscitation efforts following advanced cardiac life support (ACLS) protocols were followed, and return of spontaneous circulation (ROSC) was subsequently achieved. At the time of hospital admission, he was taken to the cardiac catheterization lab and was noted to have a near 100% occlusion of the left anterior descending (LAD) artery and a 95% occlusion of the left circumflex (LCX) artery, both requiring placement of drug-eluting stents. A 90% occlusion of the right coronary artery (RCA) was noted, but was not immediately intervened on at that time. A transthoracic echocardiogram performed identified a left atrial mass measuring 2.15 cm × 2.22 cm and was noted to be intermittently obstructing the mitral valve. The left ventricular ejection fraction (LVEF) was then noted to be 10–15% with severe diffuse hypokinesis of the left ventricle shortly after the left heart catheterization. Additionally, the right ventricle was borderline dilated and peak pulmonary pressure was 41 mmHg. The patient then became hypotensive and required an intra-aortic balloon pump, which was subsequently upgraded to an Impella device, and reached a maximum setting of p-6. He was then transferred to the intensive care unit (ICU) and placed on milrinone, amiodarone, and epinephrine intravenously as well as inhaled nitric oxide in addition to his mechanical circulatory support. His initial calculated Fick’s cardiac output and index while on mechanical and vasopressor support were found to be 6.8 L/min and 3.3 L/min/m^2^, respectively, with a venous blood gas (VBG) at the time, which had shown an oxygen saturation of 72%. Due to acute renal failure, the patient was also started on continuous renal replacement therapy.

Supplemental history was unable to be obtained as the patient was intubated and sedated at the time of arrival. A history of alcohol abuse was identified, but no other cardiac risk factors were elucidated following discussion with the patient’s family. In addition, family history was significant for bladder cancer in his brother as well as heart disease in his father.

**Fig. 1:**
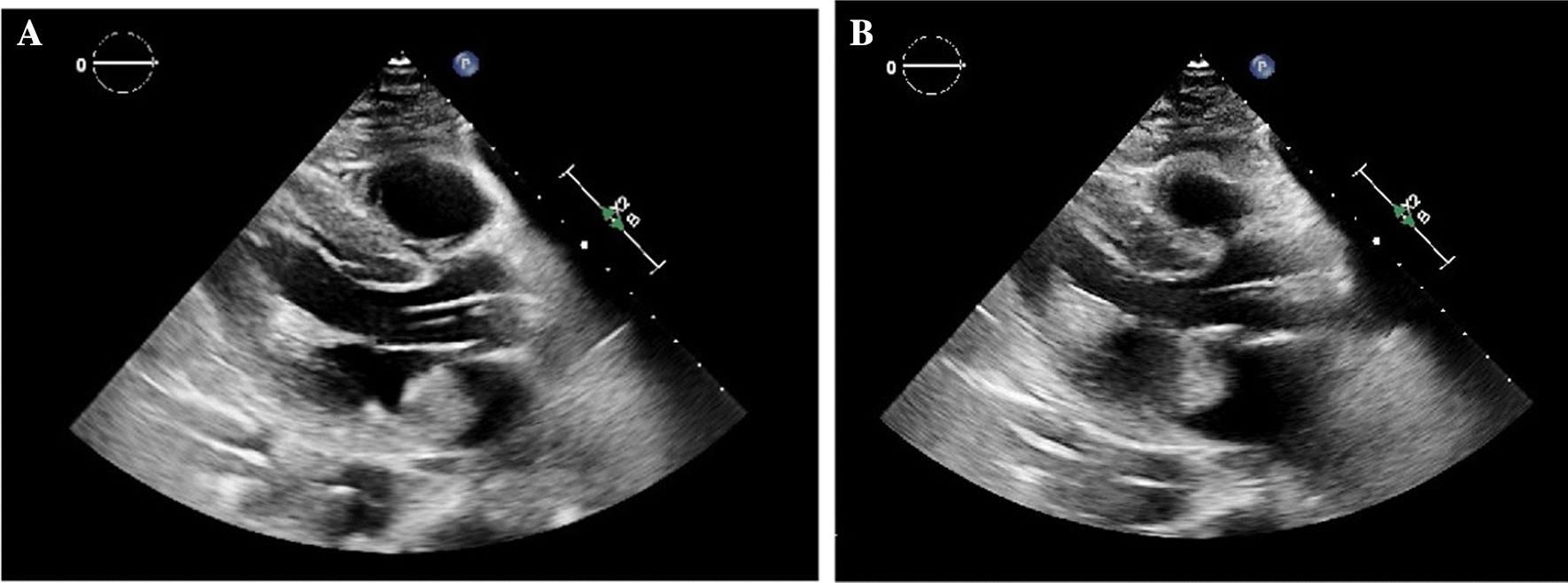
A, B: Transthoracic echocardiogram in the parasternal long axis view demonstrating a large left atrial mass intermittently obstructing the mitral valve; **A** (left image) demonstrating the left atrial mass in diastole, not obstructing the mitral valve; **B** (right image) showing the left atrial mass obstructing the mitral valve in systole

The cardiothoracic surgery and advanced heart failure teams were consulted and he was continued on hemodynamic support with multiple vasopressors and an Impella. A repeat transthoracic echocardiogram was performed 3 days following hospital presentation. LVEF was 35–40%, with diffuse hypokinesis of the left ventricular walls and the left atrial mass measuring at 3.14 cm × 2.02 cm (Fig. 1).

After the first week of hospitalization, the patient was medically optimized for a planned resection of the left atrial mass, even though he had continued to require the Impella device at p-3 for hemodynamic support. The patient was then taken to the operating room for a single-vessel coronary artery bypass graft (CABG) and excision of the left atrial myxoma. Following the procedure, the Impella was able to be removed and the left atrial myxoma was sent to pathology.

In addition, he was able to be weaned off all vasopressor and inotropic medications within a day following the procedure, with the exception of dobutamine. On postoperative day 3, he was successfully weaned off dobutamine. During this time, he was also transitioned from continuous renal replacement therapy (CRRT) to intermittent hemodialysis (iHD). He was weaned off of mechanical ventilation and was initiated on guideline-directed medical therapy (GDMT) for his acute coronary syndrome and heart failure. As the patient became medically stable, he was subsequently transferred to inpatient rehabilitation.

## Discussion

Myxomas are the most common primary cardiac tumor and tend to be villous and friable, which are associated with embolic events, or smooth and cause obstructive symptoms [1]. Obstructive symptoms from left-sided atrial myxomas typically include orthopnea, paroxysmal nocturnal dyspnea, and pulmonary hypertension; usually from left-sided heart failure and secondary pulmonary hypertension [1, 4]. Left atrial myxomas are associated with an increased risk of embolization, particularly in the central nervous system and retinal arteries as well as visceral organs including the spleen, kidneys, and adrenals [1, 5].

The most common complications from atrial myxomas include symptoms of heart failure, arrhythmias, valvular defects, thrombotic events, and infection [6, 7]. Atrial myxomas can lead to the presentation of heart failure with resultant fluid overload. Arrhythmias can occur due to either local invasion of the tumor or surgical resection [6]. Thrombotic events that occur are dependent on the location affected by the emboli, and patients are typically placed on anticoagulation [6]. These tumors can also serve as a nidus for infection, leading to endocarditis, sepsis, or disseminated intravascular coagulation [6].

As in this patient’s case, if a left atrial myxoma becomes large enough, it can obstruct blood flow through the mitral valve and lead to acute mitral dynamic stenosis, resulting in circulatory collapse [8]. This condition is due to obstruction of the left ventricle in diastole as the myxoma occludes the mitral valve [9]. Cardiac output is subsequently decreased and cannot be maintained in the normal range, resulting in cardiogenic shock [9]. Complicating matters in this patient’s case was the precipitating myocardial infarction. Multiple case reports have described circulatory collapse intra- and postoperatively in patients with left atrial myxoma. Only a small number of cases have been published since 1950 regarding sudden cardiac death secondary to an atrial myxoma [10].

The definitive treatment of myxomas that cause acute mitral dynamic stenosis is resection. Prior to resection, these patients can be managed medically with a combination of increasing the cardiac preload with intravenous fluids, use of beta blockers or calcium channel blockers to increase the diastolic filling time, and prevention of hypotension, which can worsen shock in these patients. On occasion, management of hypotension may require inotropic and vasopressor medications [11]. Our patient required an Impella device and multiple vasoactive medications that acted as a bridge for hemodynamic support prior to resection of the myxoma.

Upon hospital discharge and transfer to inpatient rehab, the patient was instructed to follow up with advanced heart failure and cardiothoracic surgery teams for follow-up care.

## Conclusion

Atrial myxomas are a common cardiac tumor and can result in complications of heart failure, cardiac arrhythmias, and valvular defects. In this patient’s case, his cardiogenic shock following cardiac arrest and revascularization was exacerbated by a left atrial myxoma causing mitral dynamic stenosis as a result of intermittent obstruction of the mitral valve. Definitive treatment requires surgical resection of the tumor with bridge therapy, including vasopressor medications and the use of intravenous fluids, beta blockers, or calcium channel blocker to increase the cardiac preload.

## Data Availability

As a case report, the patient’s individual information is not available for review, in accordance with HIPPA compliance.

## Abbreviations

ACLS: Advanced cardiac life support
CABG: Coronary artery bypass graft
CRRT: Continuous renal replacement therapy
GDMT: Guideline-directed medical therapy
EMS: Emergency medical services
iHD: Intermittent hemodialysis
LAD: Left anterior descending
LCX: Left circumflex
LVAD: Left ventricular assist device
LVEF: Left ventricular ejection fraction
RCA: Right coronary artery
